# Combined Therapeutic Strategies Based on the Inhibition of Non-Oncogene Addiction to Improve Tumor Response in EGFR- and KRAS-Mutant Non-Small-Cell Lung Cancer

**DOI:** 10.3390/cancers16233941

**Published:** 2024-11-25

**Authors:** Luisa Amato, Daniela Omodei, Caterina De Rosa, Annalisa Ariano, Sara Capaldo, Camilla Carmela Tufano, Rossella Buono, Cristina Terlizzi, Anna Nardelli, Vitale Del Vecchio, Rosanna Palumbo, Concetta Tuccillo, Floriana Morgillo, Federica Papaccio, Virginia Tirino, Francesca Iommelli, Carminia Maria Della Corte, Viviana De Rosa

**Affiliations:** 1 Department of Precision Medicine, University of Campania Luigi Vanvitelli, 80131 Naples, Italy; luisa.amato@unicampania.it (L.A.); caterina.derosa1@unicampania.it (C.D.R.); annalisa.ariano9826@gmail.com (A.A.); saracapaldo99@gmail.com (S.C.); concetta.tuccillo@unicampania.it (C.T.); floriana.morgillo@unicampania.it (F.M.); carminiamaria.dellacorte@unicampania.it (C.M.D.C.); 2 Institute of Biostructures and Bioimaging, National Research Council, 80145 Naples, Italy; daniela.omodei@ibb.cnr.it (D.O.); rossellabuono00@gmail.com (R.B.); cristina.terlizzi@ibb.cnr.it (C.T.); anna.nardelli@ibb.cnr.it (A.N.); rosanna.palumbo@cnr.it (R.P.); viviana.derosa@ibb.cnr.it (V.D.R.); 3 Department of Experimental Medicine, University of Campania Luigi Vanvitelli, 80138 Naples, Italy; camillacarmela.tufano@unicampania.it (C.C.T.); vitale.delvecchio@unicampania.it (V.D.V.); virginia.tirino@unicampania.it (V.T.); 4 Department of Life Sciences, Health and Health Professions, Link Campus University, 00165 Rome, Italy; 5 Department of Medicine, Surgery and Dentistry, “Scuola Medica Salernitana”, University of Salerno, 84084 Baronissi, Italy; fpapaccio@unisa.it; 6 Clinical Pharmacology Unit, San Giovanni di Dio e Ruggi d’Aragona University Hospital, 84131 Salerno, Italy

**Keywords:** DDR, energy metabolism, NOA therapy, NSCLC

## Abstract

Background: Oncogene-driven NSCLC is usually treated with targeted therapies using tyrosine kinase inhibitors (TKIs) to inhibit oncogene downstream signaling pathways, affecting tumor survival and proliferation. EGFR- and KRAS-mutant NSCLCs are the most represented subtypes, and they are treated in clinical practice with oncogene-targeting drugs in the first and second line, respectively. Unfortunately, the development of oncogene-independent resistant clones limits TKI efficacy. Here, we used non-oncogene addiction (NOA) as an innovative therapeutic strategy to target other essential proteins that support changes in tumor phenotype. Specifically, we tested, for the first time, a combination of inhibitors, namely ATR, involved in DNA damage response, and pyruvate dehydrogenase kinases (PDKs), involved in energy metabolism. Methods: Sensitive PC9 and the corresponding EGFR-TKI-resistant PC9/OR, EGFR-mutant H1975, and KRAS-mutant A549 NSCLC cells, were treated with TKIs (osimertinib and selumetinib, respectively). In parallel, cells were exposed to two combination regimens: one using the TKI with an ATR inhibitor and the other one combining the two selected NOA inhibitors (ATR inhibitor, M4344; and PDK inhibitor, DCA). Results: The effect of these two combined approaches, compared to TKI alone, produced similar results in terms of cell proliferation, cell death, and migration. Thus, depending on tumor biology, selecting between the proposed therapeutic strategies will be different, to maximize tumor response. Conclusions: The major translational relevance of this study is to exploit new targets for the development of innovative and improved therapeutic strategies with NOA drugs, over combinations including target genes within the oncogene pathway, to overcome resistance to TKI therapies in patients with NSCLC who are oncogene-addicted.

## Simple Summary

Oncogene-driven non-small-cell lung cancer (NSCLC) is typically treated with targeted therapies to inhibit oncogene downstream signaling pathways and reduce tumor survival. The most common subtypes, EGFR and KRAS mutations, are amenable to these therapies; however, resistance often develops, leading to oncogene-independent metastases. This study explores non-oncogene addiction (NOA) as a novel strategy, targeting essential genes like ATR, which is involved in DNA damage response, and pyruvate dehydrogenase kinases (PDKs), which play a role in energy metabolism. Experiments were conducted using sensitive PC9 and the corresponding osimertinib-resistant cells (PC9/OR), namely EGFR-mutant H1975 and KRAS-mutant A549 cells treated with TKIs, alongside ATR and DCA inhibitors. The results indicated that combining these approaches could enhance efficacy compared to TKIs alone, suggesting a tailored strategy based on tumor subtype. This research underscores the potential of new therapeutic targets to improve treatment outcomes in patients with NSCLC compared to traditional TKI therapies.

## 1. Introduction

Cancer indicates a multitude of distinct genetic diseases with common hallmarks [1,2] that, unlike normal cells, are subject to the hyperactivation of oncogenic pathways responsible for altered proliferation programs and cell death resistance to drug stimuli. The strong dependence on the oncogenic signaling of several tumors, a phenomenon called oncogene addiction (OA) [3], leads to the development of targeted therapies to inhibit the oncogenes [4]. Unfortunately, despite the initial therapeutic response, tumor regrowth will occur due to the survival of drug-resistant clones in most patients [5]. In particular, EGFR-mutant NSCLC represents the major example of oncogene addiction, and KRAS-mutant NSCLC represents the most common oncogene-driven indicator in NSCLC [6]. In this scenario, we approach the conceptual framework of non-oncogene addiction (NOA) inhibition [7] alone or in combination with oncogene-driver inhibitors and how this can be exploited to develop new innovative therapeutic approaches in NSCLC to prevent the occurrence of resistance. To face intracellular stress due to selective drug pressure, tumors become dependent on a plethora of essential genes (not mutated or aberrantly expressed) that are necessary to support the transformed phenotype, even though not directly involved in tumor initiation. In particular, NOA targets are involved in DNA damage and replication, mitotic, proteotoxic, metabolic, and oxidative stress, which are clearly interconnected with one another. The majority of cancer cells present a genomic instability that causes enhanced sensitivity to agents that interfere with this stress-response pathway; inhibitors of DDR kinases exhibit selective toxicity toward cancer cells. Moreover, the tumor-altered metabolism, called the Warburg effect, diverts resources toward biosynthetic pathways, reduces ROS production through mitochondrial oxidative phosphorylation, and copes with oxygen availability in the tumor microenvironment. Exploiting the DDR and the Warburg effect as potential therapeutic opportunities can limit the toxicity of these therapies on normally proliferating cells. Our group has previously demonstrated that EMT-related (epithelial-to-mesenchymal transition) pathways can be actively explored as targets for combination approaches in EGFR-mutant NSCLC [8]. EMT is a process by which epithelial cells gradually lose their cell–cell adhesion ability and gain migratory and invasive properties to become mesenchymal cells. More recently, we also demonstrated the relevance of targeting the DNA damage response (DDR) along with ITGB1 (a marker of EMT) to prevent and avoid resistance to oncogene-driver-targeting drugs, such as osimertinib and selumetinib, that act on EGFR/RAS/MEK pathways [9,10]. Among DDR proteins, ataxia telangiectasia and Rad3-related (ATR) has been shown as a key mediator of EMT-guided resistance through activation of BIM-induced apoptosis in EGFR-resistance [11,12].

In addition, we have already explored the potential role of the selected NOA inhibitors as an innovative treatment strategy to increase tumor response and avoid resistance. In particular, we first demonstrated a non-canonical role of PDK1 as a negative regulator of apoptosis; its silencing, indeed, is associated with an increase in BAX and a concomitant decrease in Bcl-2/Bcl-xL at the mitochondria interface in NSCLC. This pro-apoptotic status was confirmed in ex vivo shPDK1 tumors associated with an increase in BIM and cleaved lamin A/C and a decrease in HIF-1α, Bcl-2/Bcl-xL, and mutant p53 [13].

In this scenario and for the present study, we selected PDKs and ATR as novel NOA targets to be combined to efficiently target apoptosis pathways and tested the effect of NOA inhibitors alone or in combination in comparison to single oncogene-targeted inhibitors. We also investigated the combination effect of the oncogene inhibitor with the NOA inhibitor to determine whether targeting the downstream oncogene pathways is essential in combination regimens.

The aim of this study was to demonstrate that co-targeting PDKs and ATR may induce the concurrent inhibition of multiple pathways involved in cell proliferation, metabolism, and EMT, thus preventing drug resistance caused by the selective drug pressure of a targeted single agent. Furthermore, we tested the effect of NOA inhibitors used in combination as a potential strategy to overcome drug resistance in cell models where the oncogene driver is not efficiently inhibited.

In summary, we propose that a better understanding of the non-canonical role of NOA targets could represent a novel cancer Achilles’ heel, opening a new clinical approach for patients with EGFR/KRAS-mutant lung cancer.

## 2. Materials and Methods

### 2.1. Cell Lines and Treatment

The PC9, H1975, and A549 cell lines were purchased and authenticated by the American Type Culture Collection (ATCC). In particular, the PC9 cells are characterized by the deletion delE746_A750 of the kinase domain of *EGFR,* conferring sensitivity to erlotinib. H1975 cells, on the other hand, hold an activating point mutation in exon 21 (L858R) of the kinase domain of *EGFR*, which also harbors the T790M mutation, conferring resistance to erlotinib. A549 cells bear the *KRAS* mutation (G12S) and wild-type *EGFR*. In addition, to obtain osimertinib-resistant (OR) cell lines, PC9 cells were continuously exposed to osimertinib, as previously described [14]. PC9, PC9/OR, and H1975 cells were cultured in RPMI 1640 (S.I.A.L. Srl, Rome, Italy) medium, while A549 cells were cultured in DMEM (Aurogene Srl, Rome, Italy), both supplemented with 10% fetal bovine serum (FBS), 100 IU/mL penicillin, and 50 mg/mL streptomycin at 37 °C in the presence of 5% CO_2_.

### 2.2. Treatment and Cell Viability

Cells were treated with oncogene inhibitors of double-mutant EGFR^L858R/T790M^, such as osimertinib (Selleck Chemicals, Houston, TX, USA) and MEK-inhibitor selumetinib (Selleck Chemicals, Selleckchem) to target KRAS downstream signaling and vehicle for 48 h at the indicated doses. Concerning NOA targets, cells were treated with dichloroacetate (DCA, Sigma–Aldrich) as an inhibitor of PDKs, M4344 (Selleck Chemicals, Selleckchem) as an inhibitor of ATR at the indicated time and doses alone or in combination among them. In particular, cells were treated with 1 μM osimertinib, 5 μM selumetinib, 500 μM DCA, and 2 μM M4344 alone. Combined treatments were obtained with 0.5 μM osimertinib plus 1 μM M4344 for PC9, H1975, and PC9/OR cells, 2.5 μM selumetinib plus 1 μM M4344 for A549 cells, and 250 μM DCA plus 1 μM M4344 for all cell lines for 48 h. The inhibitor concentration was, in all cases, half of the respective single therapy.

An automatic cell counter (LUNA-II™ Automated Cell Counter, Logos Biosystems, South Korea) was used for cell-number evaluation; after cellular staining with trypan blue, it gave us information about the total cell number and viability.

### 2.3. MTT Assay

The viability of NSCLC cell lines following a 96 h treatment period was evaluated using MTT assay, as previously described [9]. Briefly, a total of 3000 cells were seeded in each well of a 96-plate for 96 h with TKIs, DCA, and M4344, either alone or in combination. The dose of each treatment was mentioned above. The number of viable cells was determined by measuring the absorbance at 490 nm using a spectrophotometer. The data are expressed as a percentage of viable cells, with the viability of CTRL cells set at 100%. 

### 2.4. Immunoblotting Analysis

Whole cell lysates were prepared in accordance with the previously described method. Briefly, untreated and treated cells were lysed on ice in RIPA lysis buffer (R0278, Sigma–Aldrich, St. Louis, MO, USA) with protease (P8340, Sigma–Aldrich) and phosphatase inhibitors (P0044, Sigma–Aldrich). The suspension was kept on ice for 30 min and then centrifuged at 13,000× *g* at 4 °C for 20 min. Western blot analysis of proteins from different lysates was performed. The following antibodies were used for Western blotting: α-tubulin (T9026, Sigma–Aldrich), p-EGFR^Tyr1068^ (2236, Cell Signaling Technology, Danvers, MA, USA), EGFR (sc-03, SantaCruz Biotechnology, Dallas, TX, USA), p-ERK1/2 (9106, Cell Signaling Technology), ERK1/2 (9102, Cell Signaling Technology), p-ATR^Ser428^ (2853, Cell Signaling Technology), ATR (2790, Cell Signaling Technology), p-p53^Ser15^ (9284, Cell Signaling Technology) p53 (sc-126, SantaCruz Biotechnology), p-PDH^Ser293^ (ab92696, Abcam, Cambridge, UK), PDH-E1α (D-6) (sc-377092, SantaCruz Biotechnology), GAPDH (2118, Cell Signaling Technology), p-H2AX^Ser139^ (9718, Cell Signaling Technology), H2AX (7631, Cell Signaling Technology), pChk1^Ser317^ (12302, Cell Signaling Technology), Chk1 (2360, Cell Signaling Technology), p-cyclin D1 (Thr286) (D29B3) (3300, Cell Signaling Technology), cyclin D1 (92G2) (2978, Cell Signaling Technology), BID (7A3) (2006, Cell Signaling Technology), Bcl-2 (D55G8) (4223, Cell Signaling Technology). A commercially available ECL kit (Biorad, Hercules, CA, USA cat. n. 170-5061) was used to reveal the reaction.

### 2.5. Glycolytic and Mitochondrial ATP Rates 

ATP production through glycolysis and electron transport chain (ETC) was determined using the Seahorse XFp Analyzer (Agilent Technologies, Santa Clara, CA, USA) and the real-time ATP rate assay kit (Agilent Technologies) following the manufacturer’s instructions. Briefly, all cell lines were seeded on XF 96-well microplates allowed to attach overnight and the next day treated, as described before, for 48 h. Data were collected (at least 5 independent measurements), normalized for cell number, and represented as an energetic map by reporting mitochondrial and glycolytic ATP contribution.

### 2.6. Cytofluorimetric Analysis of Cell Cycle Profile and Cell Death

For cell cycle analysis, cells were treated as previously described for 48 h, collected, washed with PBS, and fixed in 70% ice-cold ethanol. Incubation with 50 μg/mL propidium iodide (PI; cat# P4170; Sigma–Aldrich) and 1 mg/mL RNase (cat# 9001-99-4; Sigma–Aldrich) at RT was performed. The analysis was carried out with FACS CANTO II and ModFit LT. For apoptosis detection, cells were stained with Annexin V-FITC and PI (BD Pharmingen, San Diego, CA, USA) according to the manufacturer’s instructions and analyzed with FACS CANTO II. Analysis was performed by Diva software 8.

### 2.7. Mitochondrial Membrane Potential

The mitochondrial membrane potential (MMP) is a characteristic parameter indicating mitochondrial function, and when MMP is reduced, cells undergo apoptosis. The MMP can be measured using the TMRE (tetramethylrhodamine, ethyl ester) dye. Briefly, all cell lines were seeded in dark 96-well plates at the density of 5000 cells/well and treated or not with selected TKIs alone or in combination (at half doses) for 48 h as previously described. First, we incubated control cells with 50 μM CCCP (carbonyl cyanide 3-chlorophenylhydrazone) as the positive control (MMP loss) for 5 min at 37 °C and 5% CO_2_. Then, all wells were incubated with 200 nM TMRE staining solution for 20 min at 37 °C and 5% CO_2_ and then washed with PBS. For quantitative analysis, fluorescence intensity was measured at Ex/Em: 550/580 nm with a fluorescence microplate reader (VICTOR^®^ Nivo™ multimode plate reader, Revvity, Waltham, MA, USA). We also captured representative images using a high-resolution fluorescence microscope (ECLIPSE Ti2, Nikon, Tokyo, Japan). At least three independent experiments were performed.

### 2.8. Wound-Healing Assay

To monitor migration ability, we performed a wound-healing assay to follow the change in the cell-covered area (gap closure) over time. Cells were seeded in 6 well plates at a density of 300,000 cells/well and allowed to attach overnight. After 24 h, we created a physical gap within a cell monolayer (using a pipette tip), and we added the different treatments. Then, we monitored the process of cell migration into the gap by taking pictures at different time points (from T0 h to T48 h) by selecting XY plate coordinates with a high-resolution phase-contrast microscope. To analyze the gap closure rate, we manually drew the distance between the gaps, and we expressed the % of the gap remaining versus each condition at T0. At least three independent experiments were performed and pooled together.

### 2.9. Immunofluorescence (IF)

Cells (10,000/well) were seeded in 96-well plates and then treated for 48 h. Cells were then fixed for 20 min with a 4% paraformaldehyde (PFA) solution and permeabilized for 20 min with 0.3% Triton X-100 1% BSA in PBS buffer at RT. Three washes with PBS followed by incubation overnight at 4 °C with the primary antibody anti-E-cadherin (D2P2F) (Cell Signaling Technology, cat. n. 3195S, 1:1000) and anti-Vimentin (Santa Cruz Biotechnology, cat. n. sc-6260, 1:200) in antibody dilution buffer (0.1% Triton X-100 in 1% BSA PBS) followed by revelation using Alexa Fluor 488-conjugated anti-rabbit IgG antibodies and Alexa Fluor 647-conjugated anti-mouse IgG antibodies (Jackson Immunoresearch Laboratories, West Grove, PA, USA) at a dilution of 1:250 for 1 h. Nuclei were stained with DAPI (1 µg/mL) (Sigma-Aldrich). The fluorescence was analyzed by a high-resolution fluorescence microscope equipped with a 20× lens (ECLIPSE Ti2, Nikon, Japan).

### 2.10. Statistical Analysis and Graphical Elaboration

All statistical analyses were conducted using the Prism 8 software (GraphPad Software, San Diego, CA, USA). ANOVA, or unpaired Student *t*-test, was used as appropriate. A *p*-value < 0.05 was considered statistically significant. The Western blotting signals were quantified by morpho densitometric analysis using ImageJ software 1.53 k (NIH, Bethesda, MD, USA). Briefly, the product of the area and optical density of each band were determined and normalized to the same parameter derived from the equal loading used. Data were expressed as relative protein levels (fold change) of each treated sample compared to the corresponding vehicle-treated internal control.

## 3. Results

### 3.1. Effect of Selected Drugs on Cell Viability and Energetic Status in EGFR and KRAS-Mutant NSCLC Cell Lines

**Figure 1:**
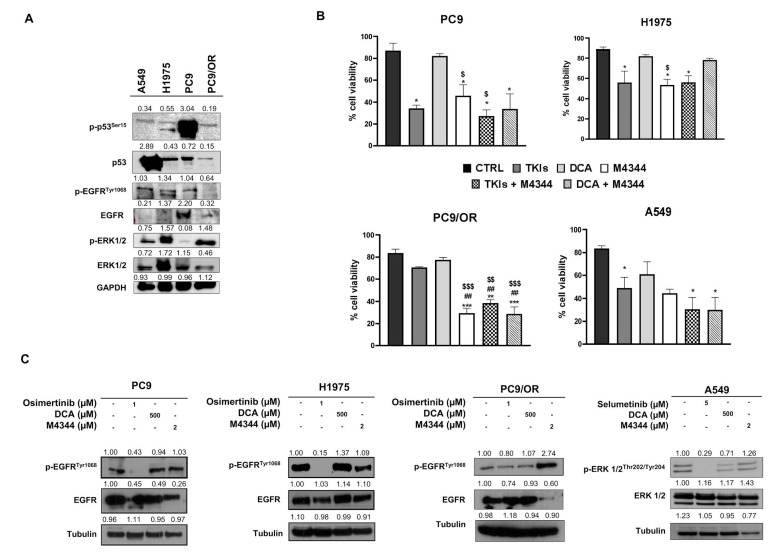
(**A**) Western blot images of whole cell lysates showed levels of phosphorylated and total forms of EGFR, ERK1/2, and p53 in whole cell lysates of selected NSCLC cell lines. (**B**) Cell viability assay of PC9, H1975, PC9/OR and A549 NSCLC cells exposed to TKI (1 μM osimertinib for PC9, H1975 and PC9/OR; 5 μM selumetinib for A549), DCA 500 μM and M4344 2 μM for 48 h alone or in combination with half the dose (TKI plus M4344 or DCA plus M4344). Data are expressed as mean ± SE. Statistical significance * *p* < 0.05, ** *p* < 0.01 and *** *p* < 0.001 versus CTRL; ^##^ *p* < 0.01 versus TKIs, ^$^ *p* < 0.05, ^$$^ *p* < 0.01 and ^$$$^ *p* < 0.001 versus DCA. At least three independent experiments were performed. (**C**) Western blot images of whole cell lysates showed levels of phosphorylated and total forms of EGFR and ERK1/2 of PC9, H1975, PC9/OR and A549 NSCLC cells exposed to TKI (1 μM osimertinib for PC9, H1975 and PC9/OR; 5 μM selumetinib for A549), DCA 500 μM and M4344 2 μM for 48 h. Tubulin was used to ensure equal loading. Uncropped western blots images are shown in Supplemetary file.

We selected EGFR-mutant NSCLC cells with activating mutations (deletion of exon 19) and with acquired resistance (T790M mutation in EGFR and MEK/MAPK cascade inactivation). In addition, the EGFR wild-type NSCLC cells exhibited mutant KRAS (G12S). PC9 and H1975 cells are sensitive to selective TKIs, in particular osimertinib for the EGFR-mutant cells and selumetinib for the MEK/MAPK hyperactive cells. We primarily analyzed the expression of the oncogene and other key oncoproteins involved in NSCLC progression in selected cells (Figure 1A). In particular, PC9 exhibited significantly higher levels of p-p53, while A549 showed higher total p53 protein expression. PC9/OR cells showed reduced phospho- and total EGFR compared to the other NSCLC cells. Finally, H1975 and PC9/OR showed higher p-ERK1/2 compared to the other selected cells. Subsequently, the sensitivity of the PC9, H1975, A549, and PC9/OR cell lines to 1 μM osimertinib, 5 μM selumetinib, 500 μM DCA, and 2 μM M4344 was preliminarily tested, and the results are shown in Figure 1B. As expected, the cell viability (%) of parental PC9, H1975, and A549 was significantly reduced after treatment with approved TKIs, whereas PC9/OR showed no sensitivity to osimertinib as previously demonstrated [9]. Interestingly, the ATR inhibitor M4344 alone, significantly affected cell viability in all selected cell lines as compared to each control, with the highest effect towards PC9/OR. This could be due to the intrinsic phenotypic characteristics acquired during the occurrence of the resistance to osimertinib. Importantly, the combined treatment of TKI and M4344 induced a significant reduction in cell viability in PC9, H1975, PC9/OR, and A549 cells. On the other hand, the DCA and M4344 combination was also able to significantly affect the cell viability of PC9, PC9/OR, and A549, with the exception of H1975 cells. We also performed an MTT assay after 96 h treatments to assess the long-term response to therapy (Supplementary Figure S1), which confirmed the 48 h results. We then performed Western blot analysis for the oncogene pathway activation analysis after treatment with the selected TKI and NOA inhibitors (Figure 1C). As expected, we found a reduction in the phosphorylated forms of direct drug targets, confirming the oncogene addiction. In particular, we found a strong reduction in p-EGFR after osimertinib treatment in PC9 and H1975, as well as in p-ERK after selumetinib treatment in A549 cells. In addition, treatment with DCA or M4344 did not affect oncogene activation in the selected cell lines, with the exception of the PC9/OR cells showing an increase in p-EGFR after treatment with M4344.

**Figure 2:**
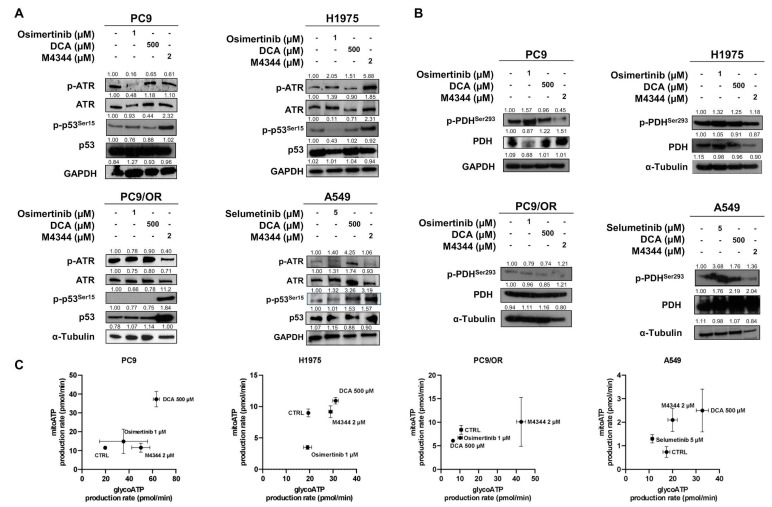
(**A**,**B**) Western blot images showed levels of phosphorylated and total forms of (**A**) ATR and p53, (**B**) PDH in the selected NSCLC cell lines treated for 48 h with TKI, DCA, and/or M4344. (**C**) Energetic map of PC9, H1975, PC9/OR, and A549 NSCLC cells exposed to TKI (1 μM osimertinib for PC9, H1975, and PC9/OR; 5 μM selumetinib for A549), DCA 500 μM and M4344 2 μM for 48 h.

We next assessed the effect of the NOA inhibitors, namely M4344 and DCA, on the selective target phosphorylation of ATR and PDH in our cell lines. In addition, we analyzed the effect of these treatments on p-p53^Ser15^ since it plays a major role in cellular response to DNA damage, and its activation can lead to either cell-cycle arrest or apoptosis [15]. Treatment with TKIs increased levels of p-ATR in H1975 and A549 cells. PC9/OR and A549 showed the highest reduction of the phosphorylated form of ATR after treatment with M4344. Treatment with DCA increased p-ATR in A549, while it did not affect ATR levels in the other cell lines. Interestingly, the treatment with M4344 caused an increase in p-p53^Ser15^ in all cells, thus indicating activation of cellular response to DNA damage (Figure 2A). We then investigated the direct metabolic targets of DCA, namely phospho- and total PDH. Figure 2B shows that treatment with TKIs affects the phosphorylation of PDH in PC9, H1975, and A549 cells. In addition, treatment with M4344 induced a reduction in p-PDH in all cell lines. Next, a Real-Time ATP Seahorse assay was performed to assess the impact of TKI and NOA inhibitors on NSCLC energy metabolism (Figure 2C). Interestingly, the different treatments affected the cell’s energetic balance. Indeed, osimertinib induced a metabolic shift towards the energetic phenotype, increasing both glycolytic and mitochondrial ATP production rates in PC9 cells, while in A549, selumetinib induced a shift towards aerobic phenotype with a reduction in glycolytic ATP rate and an increase in mitochondrial ATP production rate. For the H1975 cells, osimertinib did not change the glycolytic ATP production rate but reduced the mitochondrial one. DCA treatment shifted PC9, H1975, and A549 cell lines towards a more oxidative phenotype without affecting the ATP rate in PC9/OR. Finally, M4344 increased the glycolytic ATP production rate in PC9, H1975, and PC9/OR cells while shifting A549 cells towards mitochondrial ATP. Its effect on energy metabolism may be due to replication stress, occurring during inhibition of ATR and the mutational status of p53 [16]. These findings show a rebalance of the cellular energy metabolism induced by the tested drugs.

### 3.2. ATR and PDH Co-Targeting Effect on Cell Cycle in EGFR and KRAS-Mutant NSCLC Cell Lines

**Figure 3:**
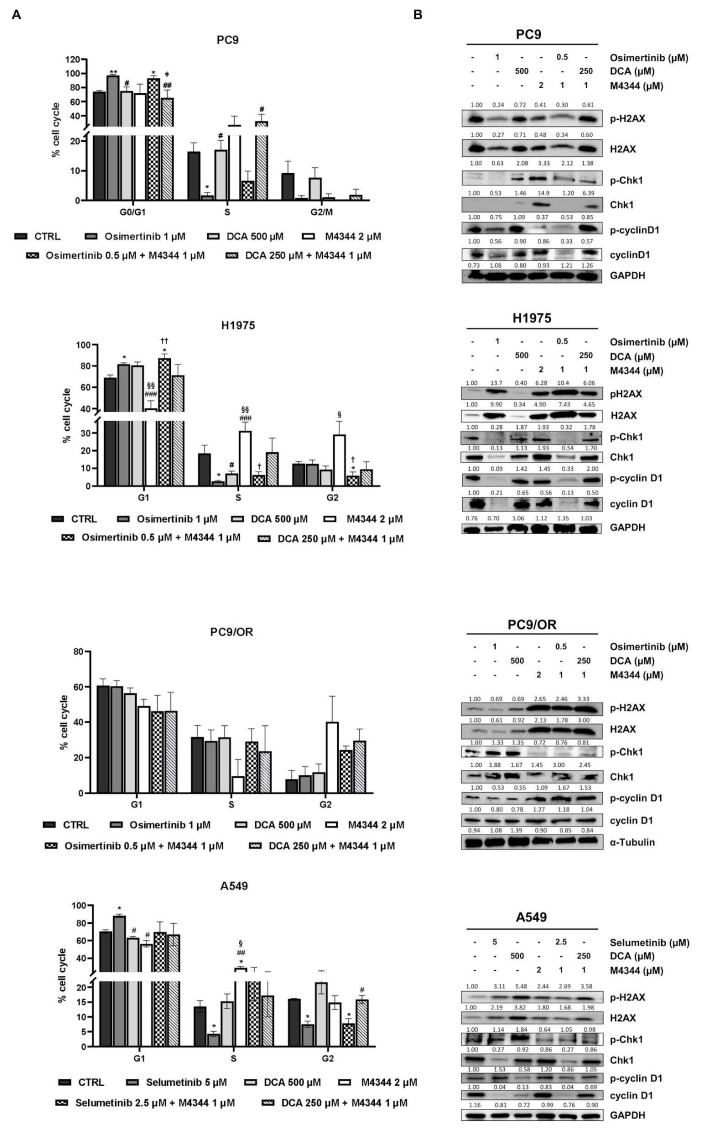
(**A**) Cell cycle analysis of PC9, H1975, PC9/OR and A549 NSCLC cells exposed to TKI (1 μM osimertinib for PC9, H1975 and PC9/OR; 5 μM selumetinib for A549), DCA 500 μM and M4344 2 μM alone or in combination at the half the dose (osimertinib/selumetinib plus M4344 or DCA plus M4344) for 48 h. Data are expressed as mean ± SE. Statistical significance * *p* < 0.05 and ** *p* < 0.01 versus CTRL; ^#^ *p* < 0.05, ^##^ *p* < 0.01 and ^###^ *p* < 0.0001 versus TKIs, ^§^ *p* < 0.05 and ^§§^ *p* < 0.01 versus DCA; ^†^ *p* < 0.05 and ^††^ *p* < 0.01 versus M4344. (**B**) Western blot images of whole cell lysates showed levels of phosphorylated and total forms of H2AX, Chk1, and cyclin D1 in selected NSCLC cell lines treated for 48 h with TKI alone or in combination with NOA inhibitors. At least three independent experiments were performed.

Next, we aimed to investigate the effect of NOA inhibitors on the cell cycle. In particular, we treated selected cells with a combination of M4344 with TKIs or with DCA at half of the concentration used for single treatments, with the aim of assessing a potentiation of the effects in combination. Figure 3A shows a significant increase of G0/G1 and a decrease in S phases after treatment with osimertinib alone or in combination in the PC9 cell line. It is interesting to note the strong reduction in the G2/M phase after all treatments except for untreated and DCA-treated conditions. In addition, osimertinib treatment alone significantly reduced the S phase in H1975. Osimertinib combined with M4344 caused a significantly increased G1 phase and reduced G2 phase in H1975 cells. Moreover, M4344 alone completely altered phase distribution, determining a G1 decrease along with an S and G2 increase compared to untreated, osimertinib- and DCA-treated samples. PC9/OR, as a model of acquired resistance to osimertinib, showed that only treatment with M4344 caused a decrease in the S phase and an increase in G2 compared to untreated, osimertinib- and DCA-treated cells. Selumetinib alone caused an increase in G1 and a decrease in S and G2 (also in combination with M4344) phases, whereas DCA did not strongly affect the cell cycle in A549 cells. Conversely, M4344 determined an opposite effect compared to selumetinib, decreasing G1 and increasing S phases. Representative curves of cell-cycle analysis were reported in Supplementary Figure S2. We also investigated whether the cell cycle was modulated by an effect on checkpoint-mediated cell cycle and DNA damage pathways by Western blot analysis. Interestingly, TKI treatment caused a strong upregulation of H2A.X and its phosphorylated form in all cell lines except for PC9/OR, whereas M4344 alone or in combination strongly increased phospho- and total H2A.X in all the selected cell lines, thus confirming that replication stress occurs as the indirect mechanism of inhibition of cell proliferation (Figure 3B). Cell-cycle checkpoints cyclin D1 and Chk1 are also modulated in response to treatments, and, in particular, p-Chk1 is reduced in response to DCA and/or M4344 in H1975, PC9/OR and A549 cells, whereas p-cyclin D1 is highly reduced after a combination of NOA inhibitors with TKI in PC9 cells. In addition, p-cyclin D1 levels were also affected after treatments with TKIs plus M4344 conditions in H1975 and A549 cells. Overall, these results indicate that the combination of TKIs with NOA targets is able to differentially affect the cell cycle in NSCLC cells.

### 3.3. Cell Death Induction in Response to Single and Combined ATR and PDH Co-Targeting 

**Figure 4:**
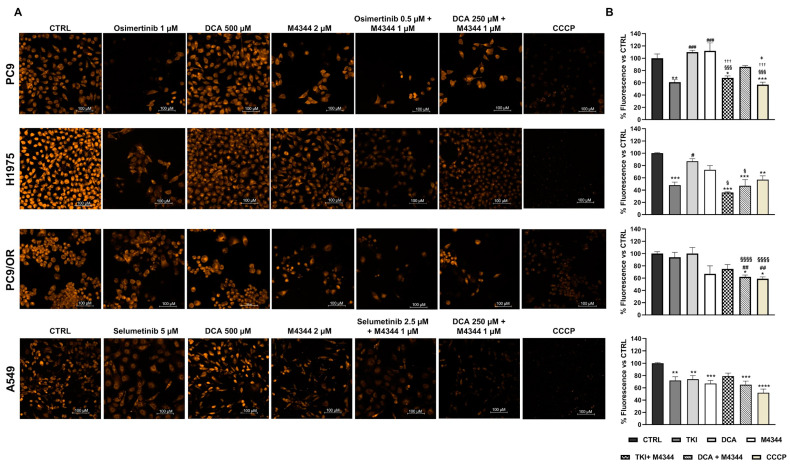
(**A**) Live images of PC9, H1975, PC9/OR, and A549 cells exposed to osimertinib (1 μM), selumetinib (5 μM), DCA (500 μM) or M4344 (2 μM) alone or in combination (at half the dose) for 48 h, or CCCP (as positive control) and stained with TMRE to visualize MMP with a high-resolution fluorescence microscope (20× magnification). (**B**) Quantitative analysis of fluorescent intensity was expressed as % relative to CTRL cells and expressed as mean ± SE. Statistical significance * *p* < 0.05, ** *p* < 0.01, *** *p* < 0.001 and **** *p* < 0.0001 versus CTRL; ^#^ *p* < 0.05, ^##^ *p* < 0.01, ^###^ *p* < 0.001 versus TKIs, ^§^ *p* < 0.05, ^§§§^ *p* < 0.001, ^§§§§^ *p* < 0.0001 versus DCA; ^†††^ *p* < 0.001 versus M4344; ^‡^ *p* < 0.05 versus DCA + M4344. At least three independent experiments were performed.

Once it was established that single or combined treatments differently affected cell cycle depending on cell line type, we aimed to investigate the mechanisms regulating cell death induction. First, we exploited MMP modulation to evaluate treatment effects on cell death by TMRE staining. MMP is a reliable indicator of mitochondrial health, and its reduction is correlated with apoptosis induction. Representative images, taken with a high-resolution fluorescence microscope, are reported in Figure 4A. As shown by quantitative analysis in Figure 4B, TKIs significantly reduced fluorescence intensity in PC9 (61%), H1975 (48%), and A549 (70%) cells, indicating an MMP decrease. M4344 alone caused a significant fluorescent decrease only in A549 cells (67%) and a slight decrease in PC9/OR (67%). Combined treatment of M4344 with TKIs increases MMP reduction in H1975 cells (36%) compared to TKI alone, thus suggesting that a TKI-combined treatment is helpful in improving cell death in a resistant cell line model. Otherwise, in a sensitive cell line model, such as PC9, TKI alone is already enough to obtain the maximum effect even if TKI-combination gave the same MMP decrease using a half dose, thus indicating that M4344 contributes to the final effect. Combined NOA treatment was less efficient in PC9 (86%) than in H1975 (47%), PC9/OR (62%), and A549 (65%) cells. These findings clearly suggest that cell lines respond differently according to their different status. In particular, the combination of TKIs + M4344 is most effective in reducing MMP in PC9 and H1975 EGFR-addicted NSCLC cells. On the other hand, the combination of M4344 + DCA is most effective in affecting MMP in PC9/OR cells and A549.

**Figure 5:**
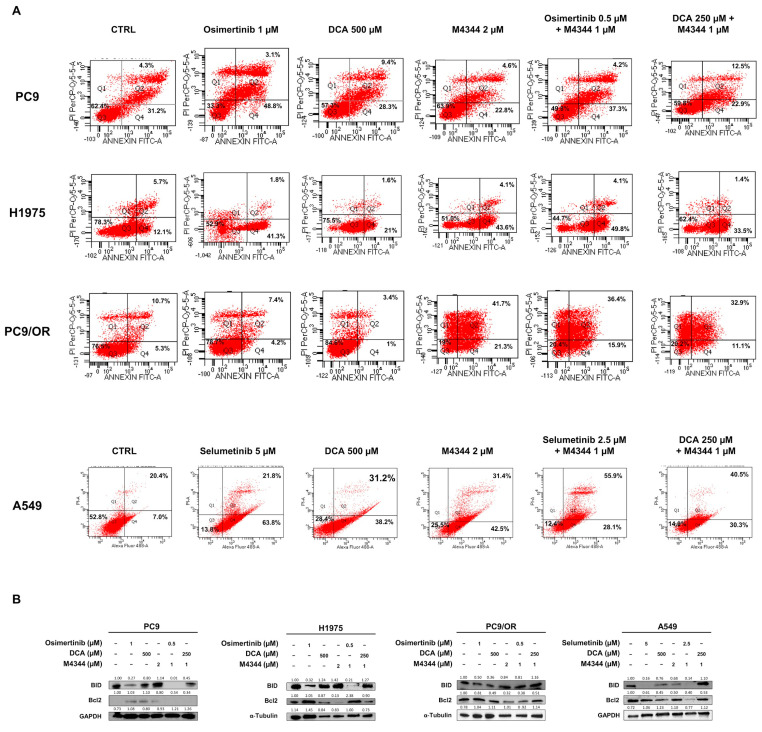
(**A**) Flow cytometry analysis of cell death by Annexin V/PI assay of PC9, H1975, PC9/OR, and A549 cells treated or not with TKIs (osimertinib 1 μM and selumetinib 5 μM), DCA (500 μM) and M4344 (2 μM) alone or in combination (at half the dose). (**B**) Representative Western blotting of whole cell lysates showing levels of BID and Bcl2 in selected NSCLC cell lines treated for 48 h with TKIs alone or combined with DCA and/or M4344. GAPDH and Tubulin were used to ensure equal loading.

In parallel, we performed Annexin V/PI apoptosis staining by flow cytometry (Figure 5A). After 48 h of treatment, we found the highest apoptotic effect in PC9 cells after treatment with osimertinib (48.8% early and 31.2% late apoptosis) whereas the other treatments caused early apoptosis of 28.3% (DCA), 22.8% (M4344), 37.3% (osimertinib + M4344) and 22.9% (DCA + M4344) and a late apoptosis of 9.4% (DCA), 4.8% (M4344), 4.2% (osimertinib + M4344), 12.5% (DCA + M4344). H1975 cells showed strong induction of early apoptosis after treatment with osimertinib (41.3%) and M4344 (43.6%), whereas DCA induced only 21% of early apoptosis. Similar results were obtained after combined treatments. As expected, apoptosis was not affected by osimertinib and DCA treatment in PC9/OR cells, whereas, interestingly, M4344 caused 21.3% of early and 41.7% of late apoptosis. Despite the inefficiency of osimertinib and DCA alone, both combined treatments caused a similar induction of early (~13%) and late (~34%) apoptosis in these cells. Conversely, selumetinib treatment induced 63.8% of early and 21.8% of late apoptosis, whereas DCA and M4344 approximately 40% of early and 30% of late apoptosis in A549 cells. Unexpectedly, both combined treatments shifted the apoptotic balance from the early to the late apoptosis. In particular, the early apoptosis percentage was 30%; instead, the late apoptosis percentage was 55.9% after treatment with selumetinib plus M4344, and it was 40.5% after treatment with DCA plus M4344. These results confirmed the viability assay findings, showing that the best response is dependent on the biological status of selected cell lines. Specifically, TKI-sensitive cell lines (PC9 and H1975) showed early and late apoptosis under single and combined treatments, while cell lines that are less sensitive to TKI (PC9/OR and A549) showed stronger activation of late apoptosis under combination. In addition, we evaluated the expression levels of two proteins of the Bcl2 family (Figure 5B). Pro-apoptotic BID protein was strongly increased with NOA target alone or in combination in PC9 and A549 cells. Importantly, treatment with TKI alone did not affect BID upregulation. Anti-apoptotic Bcl2 protein was highly reduced in DCA, M4344, and osimertinib plus M4344 in H1975 cells. PC9/OR showed only a slight decrease of Bcl2 after treatment with M4344. A strong reduction of Bcl2 was observed in selumetinib and M4344 conditions in A549 cells.

Taken together, these data clearly indicate that co-targeting of PDKs and ATR is able to increase the pro-apoptotic effect of single agents in EGFR/KRAS dependent models; specifically, selected cell lines showed a different response in terms of cell death depending on their different biological properties and phenotypes. 

### 3.4. ATR and PDH Co-Targeting Effect on Migration Ability and EMT Features 

**Figure 6:**
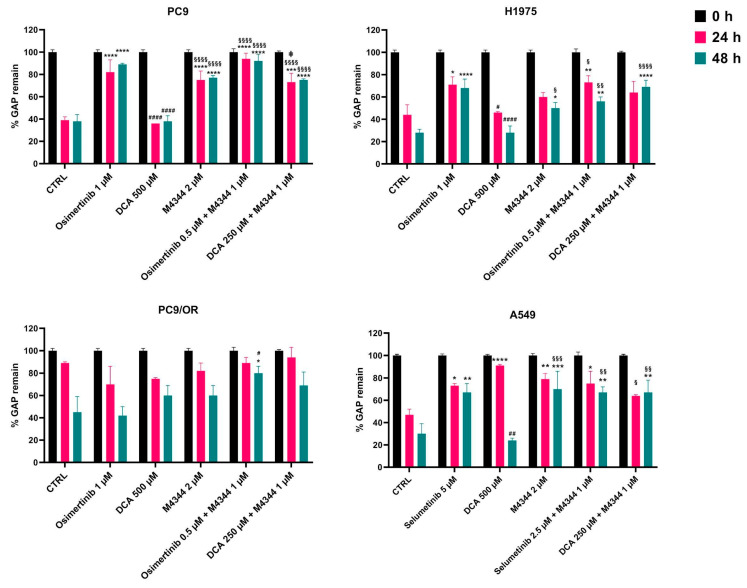
Quantitative analysis of wound-healing assay of PC9, H1975, PC9/OR, and A549 cells treated or not with TKIs (osimertinib 1 μM and selumetinib 5 μM), DCA (500 μM) and M4344 (2 μM) alone or in combination (at half the dose) from time 0 to 48 h. Histogram bars represent the percentage of gap remaining reported versus each condition at T0 as mean ± SE. Statistical significance * *p* < 0.05, ** *p* < 0.01, *** *p* < 0.001, **** *p* < 0.0001 versus CTRL; ^#^ *p* < 0.05 and ^##^ *p* < 0.001 and ^####^ *p* < 0.0001 versus TKIs, ^§^ *p* < 0.05, ^§§^ *p* < 0.01, ^§§§^ *p* < 0.001 and ^§§§§^ *p* < 0.0001 versus DCA; ^‡^ *p* < 0.05 versus DCA + M4344. At least three independent experiments were performed.

We previously demonstrated that a concomitant activation of EMT with increased cell migration ability occurs even if TKI treatments increase cell death and decrease proliferation [17]. This may be due to the heterogeneity of tumors and the presence of different cell subpopulations. In this context, we exploited the effect of combined treatments on cell migration through wound-healing assay over time (T0-T48). Figure 6 shows the quantitative percentage of gaps remaining after treatments. In particular, osimertinib alone or in combination with M4344 showed a remaining gap of 89% and 92% after 48 h in PC9 cells compared to untreated (38%) and DCA-treated (38%) cells. M4344 alone or in combination with DCA causes a mid-remaining gap of 77% and 75%. Interestingly, the significant difference between the two combination approaches indicated that OA-targeting inhibits cell spreading to a higher degree than NOA-targeting in PC9. In H1975 cells, the largest gap was observed with osimertinib (68%) and DCA plus M4344 (69%), whereas osimertinib plus M4344 treatment left a 56% gap after 48 h. In this model of acquired resistance, targeting multiple concomitant pathways using NOA treatments is necessary to reduce cell migration. As expected, PC9/OR single treatments did not affect cell migration ability compared to control. Otherwise, osimertinib or DCA plus M4344 kept the gap open at 80% and 69%, respectively. Selumetinib, M4344, and both combined treatments left a gap of approximately 69% after 48 h in A549. Representative images of initial and final time points of all cell lines and treatments visually confirmed quantitative analysis and are reported in Supplementary Figure S3. These findings confirmed that the combination reduced cell migration ability compared to single treatments.

**Figure 7:**
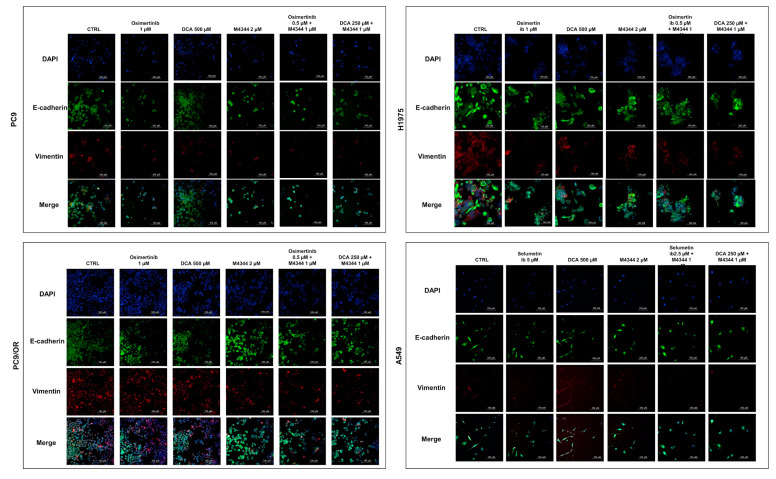
Representative immunofluorescence images of PC9, H1975, PC9/OR, and A549 cells exposed to osimertinib (1 μM), selumetinib (5 μM), DCA (500 μM) or M4344 (2 μM) alone or in combination (at half the dose) for 48 h. Nuclei were stained with DAPI (blue), and colocalization of e-cadherin (green) and vimentin (red) was observed with a high-resolution fluorescence microscope (20× magnification). Merge images were also obtained.

In parallel, we evaluated the expression of two epithelial and mesenchymal markers by immunofluorescence (IF) (Figure 7 and Supplementary Figure S4). In PC9, H1975, and PC9/OR, both e-cadherin and vimentin were expressed in basal conditions, while vimentin was not detectable in A549 cells, probably due to their high epithelial phenotype. Osimertinib treatment significantly reduced vimentin expression in PC9 e H1975 cells (0.74 and 0.58-fold, respectively) along with M4344 (0.8 and 0.60-fold, respectively), while DCA (1.12 and 0.72-fold, respectively) caused an opposite effect compared to CTRL cells. As with osimertinib, a decrease of vimentin was observed in both combination treatments in PC9 and H1975 cells (approximately 0.7 and 0.6-fold, respectively). Conversely, PC9/OR cells displayed a higher mesenchymal phenotype compared to the other cell lines based on vimentin staining. M4344 and combination treatments induced a strong decrease of vimentin (approximately 0.6-fold) compared to CTRL, osimertinib, and DCA conditions. E-cadherin and vimentin expression were not significantly affected after treatments in A549 cells. We observed a significant decrease of e-cadherin in all treatment conditions compared to untreated cells (approximately 0.76-fold) only in H1795 cells.

## 4. Discussion

The present study has been developed with the aim of exploring alternative therapeutic strategies for EGFR- and K-Ras/MEK/MAPK-driven NSCLC tumors. Currently, the EGFR-TKI osimertinib is used as the standard first-line treatment for EGFR-mutant NSCLC in clinical practice and, recently, the KRAS-G12C inhibitor sotorasib has been approved worldwide as second-line treatment for KRAS-G12C mutant NSCLC [18,19]. On one side, EGFR targeting can be considered to be the highest example of oncogene addiction in NSCLC; currently, it is clear that the majority of EGFR activating mutations can be efficiently treated with the third-generation EGFR-TKI osimertinib [20], which, however, develops resistance mechanisms through MET activation, EMT features and alterations in cell-cycle and DDR-related proteins [21]. Recently, a novel strategy using a combination of amivantamab (EGFR/MET antibody) and lazertinib (another third-generation EGFR-TKI) [22] has paved the way to clinical approval for EGFR-mutant NSCLC, thus highlighting the need to explore NOA targets to prevent resistance to RTK-mediated inhibition. On the other hand, the role of KRAS as an oncogene is different, and the role of various types of KRAS mutations is still unknown in terms of response to non-selective KRAS inhibitors [23]. In this respect, while various KRAS inhibitors are still under clinical evaluation, showing heterogeneous responses in patients, which may be due to concurrent co-mutations, novel data on KRAS resistance mechanisms are emerging [24,25]. Thus, although EGFR and KRAS-mutant NSCLC represent in clinical scenarios two different subtypes, their pathways are biologically connected: co-targeting of EGFR receptor and downstream mediators belonging to RAS/MEK signaling has shown promising efficacy in pre-clinical and clinical studies in EGFR-mutant tumors [8,26], and resistance mechanisms to EGFR/KRAS targeting may overlap. 

**Figure 8:**
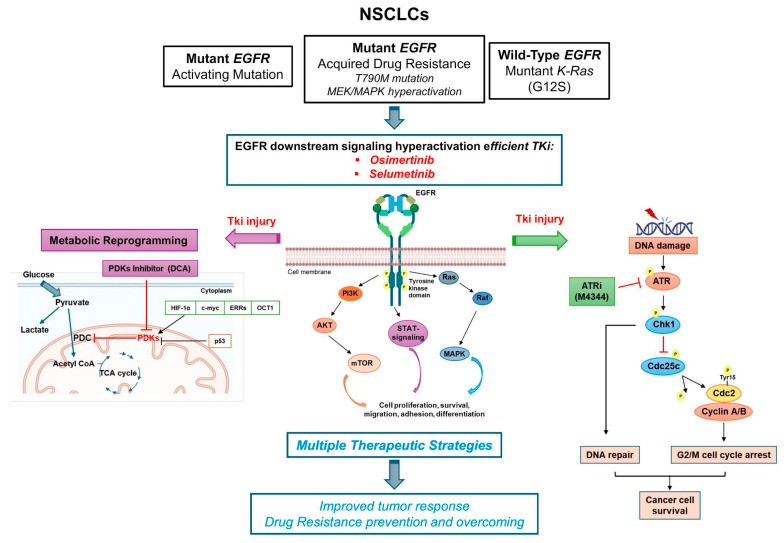
Working model of NSCLC cells based on oncogene-driven mutations and non-oncogene addiction. The graphical summary was produced by the authors.

In this context, we selected cell models of EGFR and KRAS-mutant NSCLC to study combinations of NOA targets as an alternative strategy to overcome and prevent TKI resistance, exploring the differences in various models (e.g., EGFR-TKI-sensitive versus EGFR-resistant model) as summarized in Figure 8. The opportunity to target multiple parallel pathways improves tumor response, not only in oncogene-driven cancer cells but especially in TKI-resistant cells and for those tumors where the oncogene is undruggable or even absent. 

Specifically, based on the well-known effect of the selective drug pressure of efficient TKI on favoring the occurrence of resistance due to the activation of several independent processes, we showed that both EGFR-inhibitor osimertinib and MEK-inhibitor selumetinib induced a concurrent activation of DNA damage pathways as demonstrated by p-ATR/ATR and p-H2AX/H2AX upregulation and a more oxidative metabolic phenotype. We proposed that simultaneous activation of intracellular pathways affecting cell death and proliferation programs, as well as energetic balance, may induce the selection of resistant clones with completely different biological properties than original tumor populations. Such clones, as reported by our previous studies [9], show a more aggressive phenotype as demonstrated by the occurrence of EMT, integrin β1 downstream signaling activation, and resistance to cell death mediated by impaired DDR pathways upregulation. DDR signaling includes a variety of pathways that may be altered dynamically under selective drug pressure and this is a hot topic of actual research in various types of lung cancers [27,28,29]. In TKI-resistant clones, an additional and relevant activated property is their altered metabolic program, which can be efficiently targeted with PDK inhibitors that are able to reverse the Warburg effect in oncogene-driven cells and increase the occurrence of the citric acid cycle to produce energy [13]. 

Due to this evidence, we designed innovative combinations with ATR and PDK inhibitors (M4344 and DCA, respectively) selected among all the known NOA targets [30] based on their commonly known activity on activating apoptosis in EGFR-resistant models. In particular, it is interesting to note that we found ATR inhibitor alone or in combination with TKI/DCA as the most efficient partner drug in all selected cells, whereas the PDK inhibitor shows the highest effect in PC9/OR and A549, which shows RAS/MEK/MAPK signaling upregulation. These data align with our previous paper [9], showing that MEK inhibition with selumetinib in PC9/OR is not sufficient to overcome osimertinib resistance and that the addition of DDR inhibitors (AURK or ATR inhibitor) is more effective. Interestingly, Tanaka et al. demonstrated that ATR/AURKB targeting has pro-apoptotic effects in these models [12]. We supposed that the apoptotic response in each analyzed cell line is strongly dependent on the mutation types present in the EGFR kinase domain and KRAS, as well as on the mechanism of acquired resistance to EGFR TKIs. In particular, it is interesting to note that A549 and PC9/OR cells showed a higher percentage of early apoptotic cells upon combined treatments than PC9 and H1975 cells. This can probably be explained considering that the hyperactivation of the RAS/MEK/MAPK signaling in such cells caused the development of cell populations that, under drug pressure, become more resistant to cell death. However, additional studies are needed to better understand the biochemical mechanisms of each cell line enrolled in the studies and responsible for the activation of different cell death programs upon drug injury.

In the present manuscript, we also demonstrate that co-targeting of NOA, without keeping the TKI, is able to affect the main properties of TKI resistance, inducing reduction of cell viability and migration ability, cell-cycle arrest, and EMT inhibition in both EGFR- and KRAS-mutant NSCLC models. In this respect, we are aware of the limit of the present study in the investigation of DDR pathways, since we focused on ATR signaling targeting. However, ATR inhibitors are advancing fast in clinical development [31], and there is a strong biological rationale supporting ATR from previous literature showing that ATR inhibition modulates apoptosis in EGFR-resistant models and affects KRAS-driven replication stress [32]. Future studies will clarify the appropriate single and combined regimens in animal models to confirm our in vitro results, thus identifying possible potential biomarkers to be screened in patients with NSCLC.

## 5. Conclusions

In conclusion, the findings of the present study support combinations of DDR, like ATR, with PDKs as ideal novel targets for TKI-resistant cells. Future studies could better explore biomarkers for using alternative DDR, such as ATM, AURK, or DNA-PK, in different models of NSCLC, exploring the evolution of co-mutations in resistant clones. Finally, we suggest that the design of future studies using novel combinations of NOA targets could optimize the sequence and personalization of treatments for EGFR- and KRAS-mutant NSCLC patients.

## Data Availability

The majority of data related to presented results are included in the materials and methods section of the paper.

## Supplementary Materials

The following supporting information can be downloaded at: https://www.mdpi.com/article/10.3390/cancers16233941/s1. Supplementary Figure S1. MTT assay of PC9, H1975, PC9/OR, and A549 NSCLC cells exposed to TKI (1 μM osimertinib for PC9, H1975, and PC9/OR; 5 μM selumetinib for A549), DCA 500 μM and M4344 2 μM for 96 h alone or in combination with half the dose (TKI plus M4344 or DCA plus M4344). Data are expressed as mean ± SD. Statistical significance * *p* < 0.05, ** p < 0.01 and *** *p* < 0.001 versus CTRL; ^#^ *p*< 0.05, ^##^ *p* < 0.01 versus TKIs; ^$$^ *p* < 0.01 versus DCA. At least three independent experiments were performed. Supplementary Figure S2: Representative curves of PC9, H1975, PC9/OR, and A549 cell cycle after 48 h treatments (osimertinib 1 μM, selumetinib 5 μM, DCA 500 μM or M4344 2 μM alone or in combination at half the dose). Supplementary Figure S3: Representative live images of PC9, H1975, PC9/OR, and A549 cells after scratch and treatments (osimertinib 1 μM, selumetinib 5 μM, DCA 500 μM or M4344 2 μM alone or in combination at half the dose) with a high-resolution microscope. Pictures (4× magnification) are captured by fixing XY coordinates in order to measure the same gap point over time (T0–T48). Supplementary Figure S4: Quantitative IF analysis of e-cadherin and vimentin in PC9, H1975, PC9/OR, and A549 cells after 48 h treatments (osimertinib 1 μM, selumetinib 5 μM, DCA 500 μM or M4344 2 μM alone or in combination at half the dose). Quantitative analysis of fluorescent intensity was expressed as fold change versus CTRL cells and expressed as mean ± SE. Statistical significance * *p* < 0.05, ** *p* < 0.01, *** *p* < 0.001 and **** *p* < 0.0001 versus CTRL; ^##^ *p* < 0.01, ^####^ *p* < 0.0001 versus TKIs, ^§^ *p* < 0.05, ^§§^ *p* < 0.01, ^§§§^ *p* < 0.001, ^§§§§^ *p* < 0.0001 versus DCA. At least three independent experiments were performed; Supplementary figure S5-S6-S7-S8-S9: Uncropped Western Blots.

## References

[1] Hanahan D., Weinberg R.A. (2011). Hallmarks of cancer: The next generation. Cell.

[2] Hanahan D., Weinberg R.A. (2000). The hallmarks of cancer. Cell.

[3] Weinstein I.B., Joe A. (2008). Oncogene addiction. Cancer Res..

[4] Bedard P.L., Hyman D.M., Davids M.S., Siu L.L. (2020). Small molecules, big impact: 20 years of targeted therapy in oncology. Lancet.

[5] Sabnis A.J., Bivona T.G. (2019). Principles of Resistance to Targeted Cancer Therapy: Lessons from Basic and Translational Cancer Biology. Trends Mol. Med..

[6] Morgillo F., Della Corte C.M., Fasano M., Ciardiello F. (2016). Mechanisms of resistance to EGFR-targeted drugs: Lung cancer. ESMO Open.

[7] Solimini N.L., Luo J., Elledge S.J. (2007). Non-oncogene addiction and the stress phenotype of cancer cells. Cell.

[8] Della Corte C.M., Ciaramella V., Cardone C., La Monica S., Alfieri R., Petronini P.G., Malapelle U., Vigliar E., Pepe F., Troncone G. (2018). Antitumor Efficacy of Dual Blockade of EGFR Signaling by Osimertinib in Combination With Selumetinib or Cetuximab in Activated EGFR Human NCLC Tumor Models. J. Thorac. Oncol..

[9] De Rosa C., De Rosa V., Tuccillo C., Tirino V., Amato L., Papaccio F., Ciardiello D., Napolitano S., Martini G., Ciardiello F. (2024). ITGB1 and DDR activation as novel mediators in acquired resistance to osimertinib and MEK inhibitors in EGFR-mutant NSCLC. Sci. Rep..

[10] Terlizzi C., De Rosa V., Iommelli F., Pezone A., Altobelli G., Maddalena M., Dimitrov J., De Rosa C., Della Corte C., Avvedimento V. (2023). ATM inhibition blocks glucose metabolism and amplifies the sensitivity of resistant lung cancer cell lines to oncogene driver inhibitors. Cancer Metab..

[11] Blaquier J.B., Ortiz-Cuaran S., Ricciuti B., Mezquita L., Cardona A.F., Recondo G. (2023). Tackling Osimertinib Resistance in EGFR-Mutant Non-Small Cell Lung Cancer. Clin. Cancer Res..

[12] Tanaka K., Yu H.A., Yang S., Han S., Selcuklu S.D., Kim K., Ramani S., Ganesan Y.T., Moyer A., Sinha S. (2021). Targeting Aurora B kinase prevents and overcomes resistance to EGFR inhibitors in lung cancer by enhancing BIM- and PUMA-mediated apoptosis. Cancer Cell.

[13] De Rosa V., Iommelli F., Terlizzi C., Leggiero E., Camerlingo R., Altobelli G.G., Fonti R., Pastore L., Del Vecchio S. (2021). Non-Canonical Role of PDK1 as a Negative Regulator of Apoptosis through Macromolecular Complexes Assembly at the ER-Mitochondria Interface in Oncogene-Driven NSCLC. Cancers.

[14] Della Corte C.M., Malapelle U., Vigliar E., Pepe F., Troncone G., Ciaramella V., Troiani T., Martinelli E., Belli V., Ciardiello F. (2017). Efficacy of continuous EGFR-inhibition and role of Hedgehog in EGFR acquired resistance in human lung cancer cells with activating mutation of EGFR. Oncotarget.

[15] Hernández Borrero L.J., El-Deiry W.S. (2021). Tumor suppressor p53: Biology, signaling pathways, and therapeutic targeting. Biochim. Biophys. Acta Rev. Cancer.

[16] Kang T.H. (2023). DNA Damage, Repair, and Cancer Metabolism. Int. J. Mol. Sci..

[17] Iommelli F., De Rosa V., Terlizzi C., Fonti R., Camerlingo R., Stoppelli M.P., Stewart C.A., Byers L.A., Piwnica-Worms D., Del Vecchio S. (2021). A Reversible Shift of Driver Dependence from EGFR to Notch1 in Non-Small Cell Lung Cancer as a Cause of Resistance to Tyrosine Kinase Inhibitors. Cancers.

[18] Hendriks L.E., Kerr K.M., Menis J., Mok T.S., Nestle U., Passaro A., Peters S., Planchard D., Smit E.F., Solomon B.J. (2023). Oncogene-addicted metastatic non-small-cell lung cancer: ESMO Clinical Practice Guideline for diagnosis, treatment and follow-up. Ann. Oncol..

[19] de Langen A.J., Johnson M.L., Mazieres J., Dingemans A.C., Mountzios G., Pless M., Wolf J., Schuler M., Lena H., Skoulidis F. (2023). Sotorasib versus docetaxel for previously treated non-small-cell lung cancer with KRAS(G12C) mutation: A randomised, open-label, phase 3 trial. Lancet.

[20] Eide I.J.Z., Stensgaard S., Helland Å., Ekman S., Mellemgaard A., Hansen K.H., Cicenas S., Koivunen J., Grønberg B.H., Sørensen B.S. (2022). Osimertinib in non-small cell lung cancer with uncommon EGFR-mutations: A post-hoc subgroup analysis with pooled data from two phase II clinical trials. Transl. Lung Cancer Res..

[21] Gomatou G., Syrigos N., Kotteas E. (2023). Osimertinib Resistance: Molecular Mechanisms and Emerging Treatment Options. Cancers.

[22] Cho B.C., Lu S., Felip E., Spira A.I., Girard N., Lee J.S., Lee S.H., Ostapenko Y., Danchaivijitr P., Liu B. (2024). Amivantamab plus Lazertinib in Previously Untreated EGFR-Mutated Advanced NSCLC. N. Engl. J. Med..

[23] O’Sullivan É., Keogh A., Henderson B., Finn S.P., Gray S.G., Gately K. (2023). Treatment Strategies for KRAS-Mutated Non-Small-Cell Lung Cancer. Cancers.

[24] Canon J., Rex K., Saiki A.Y., Mohr C., Cooke K., Bagal D., Gaida K., Holt T., Knutson C.G., Koppada N. (2019). The clinical KRAS(G12C) inhibitor AMG 510 drives anti-tumour immunity. Nature.

[25] Nokin M.J., Mira A., Patrucco E., Ricciuti B., Cousin S., Soubeyran I., San José S., Peirone S., Caizzi L., Vietti Michelina S. (2024). RAS-ON inhibition overcomes clinical resistance to KRAS G12C-OFF covalent blockade. Nat. Commun..

[26] Yang J.C., Ohe Y., Chiu C.H., Ou X., Cantarini M., Jänne P.A., Hartmaier R.J., Ahn M.J. (2022). Osimertinib plus Selumetinib in EGFR-Mutated Non-Small Cell Lung Cancer After Progression on EGFR-TKIs: A Phase Ib, Open-Label, Multicenter Trial (TATTON Part B). Clin. Cancer Res..

[27] Venugopala K.N. (2022). Targeting the DNA Damage Response Machinery for Lung Cancer Treatment. Pharmaceuticals.

[28] Ramkumar K., Stewart C.A., Cargill K.R., Della Corte C.M., Wang Q., Shen L., Diao L., Cardnell R.J., Peng D.H., Rodriguez B.L. (2021). AXL Inhibition Induces DNA Damage and Replication Stress in Non-Small Cell Lung Cancer Cells and Promotes Sensitivity to ATR Inhibitors. Mol. Cancer Res..

[29] Della Corte C.M., Gay C.M., Byers L.A. (2019). Beyond chemotherapy: Emerging biomarkers and therapies as small cell lung cancer enters the immune checkpoint era. Cancer.

[30] Chang H.R., Jung E., Cho S., Jeon Y.J., Kim Y. (2021). Targeting Non-Oncogene Addiction for Cancer Therapy. Biomolecules.

[31] Yano K., Shiotani B. (2023). Emerging strategies for cancer therapy by ATR inhibitors. Cancer Sci..

[32] Igarashi T., Mazevet M., Yasuhara T., Yano K., Mochizuki A., Nishino M., Yoshida T., Yoshida Y., Takamatsu N., Yoshimi A. (2023). An ATR-PrimPol pathway confers tolerance to oncogenic KRAS-induced and heterochromatin-associated replication stress. Nat. Commun..

