# Supplementary material for: Combined Therapeutic Strategies Based on the Inhibition of Non-Oncogene Addiction to Improve Tumor Response in EGFR- and KRAS-Mutant Non-Small-Cell Lung Cancer
Source: Cancers (Basel). 2024 Nov 25;16(23):3941. doi: 10.3390/cancers16233941 (PMC11639923; doi:10.3390/cancers16233941)
Supplement: Supplementary file 1 [file cancers-16-03941-s001.zip › cancers-3300854-supplementary.pdf]

Supplementary Materials for

Combined therapeutic strategies based on the inhibition of non-oncogene addiction to improve tumor response in EGFR and KRAS mutant NSCLC

Luisa Amato[1,†] , Daniela Omodei[2,†], Caterina De Rosa[1], Annalisa Ariano[1], Sara Capaldo[1], Camilla Carmela Tufano[3], Rossella Buono[2], Cristina Terlizzi[2], Anna Nardelli[2], Vitale Del Vecchio[3], Rosanna Palumbo[2], Concetta Tuccillo[1], Floriana Morgillo[1], Federica Papaccio[4,5], Virginia Tirino[3], Francesca Iommelli[2,*], Carminia Maria Della Corte[1,‡] and Viviana De Rosa[2,‡]

[1] Department of Precision Medicine, University of Campania Luigi Vanvitelli, Italy; luisa.amato@unicampania.it (L.A.); caterina.derosa1@unicampania.it (C.D.R.); annalisa.ariano9826@gmail.com (A.A.); saracapaldo99@gmail.com (S.C.); concetta.tuccillo@unicampania.it (C.T.); floriana.morgillo@unicampania.it (F.M.); carminiamaria.dellacorte@unicampania.it (C.M.D.C.)

[2] Institute of Biostructures and Bioimaging, National Research Council, Naples, Italy; daniela.omodei@ibb.cnr.it (D.O.); rossellabuono00@gmail.com (R.B.); cristina.terlizzi@ibb.cnr.it (C.T.); anna.nardelli@ibb.cnr.it (A.N.); rosanna.palumbo@cnr.it (R.P.); francesca.iommelli@ibb.cnr.it (F.I.); viviana.derosa@ibb.cnr.it (V.D.R.)

[3] Department of Experimental Medicine, University of Campania Luigi Vanvitelli, Italy; camillacarmela.tufano@unicampania.it (C.C.T.); vitale.delvecchio@unicampania.it (V.D.V.); virginia.tirino@unicampania.it (V.T.)

[4] Department of Medicine, Surgery and Dentistry, "Scuola Medica Salernitana", University of Salerno; Baronissi, Italy; fpapaccio@unisa.it

[5] Clinical Pharmacology Unit, San Giovanni di Dio e Ruggi d'Aragona University Hospital, 84131 Salerno, Italy;

The PDF file includes:

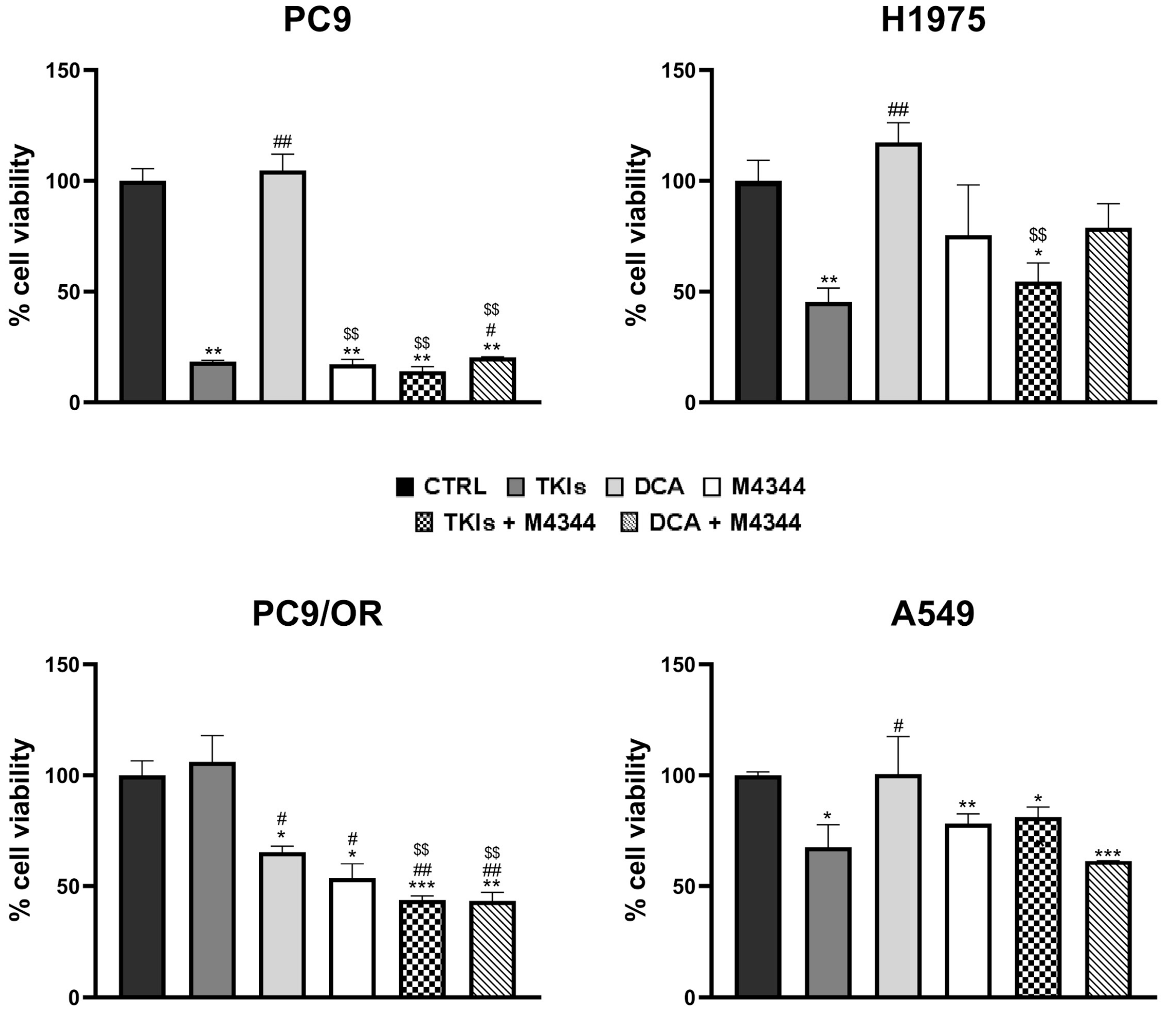

Figure S1. MTT assay of PC9, H1975, PC9/OR and A549 NSCLC cells exposed to TKI (1 μM osimertinib for PC9, H1975 and PC9/OR; 5 μM selumetinib for A549), DCA 500 μM and M4344 2 μM for 96 h alone or in combination with half the dose (TKI plus M4344 or DCA plus M4344). Data are expressed as mean ± SD. Statistical significance $^{*}p < 0.05$, $^{**}p < 0.01$ and $^{***}p < 0.001$ versus CTRL; $^{\#}p < 0.05$, $^{\#\#}p < 0.01$ versus TKIs; $^{\$\$}p < 0.01$ versus DCA. At least three independent experiments were performed.

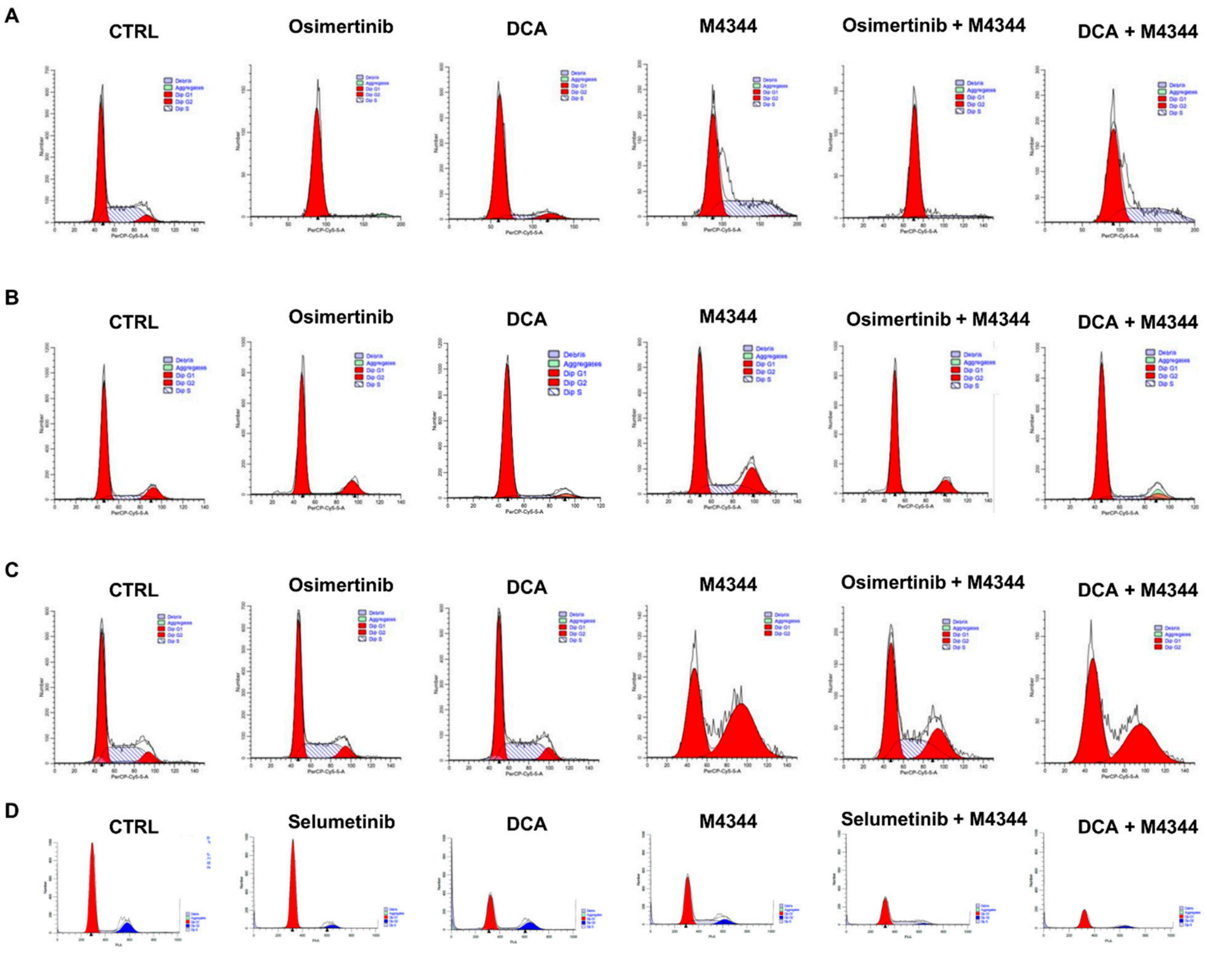

Figure S2. Representative curves of of PC9, H1975, PC9/OR and A549 cell cycle after 48 h treatments (osimertinib 1 μM, selumetinib 5 μM, DCA 500 μM or M4344 2 μM alone or in combination at half the dose).

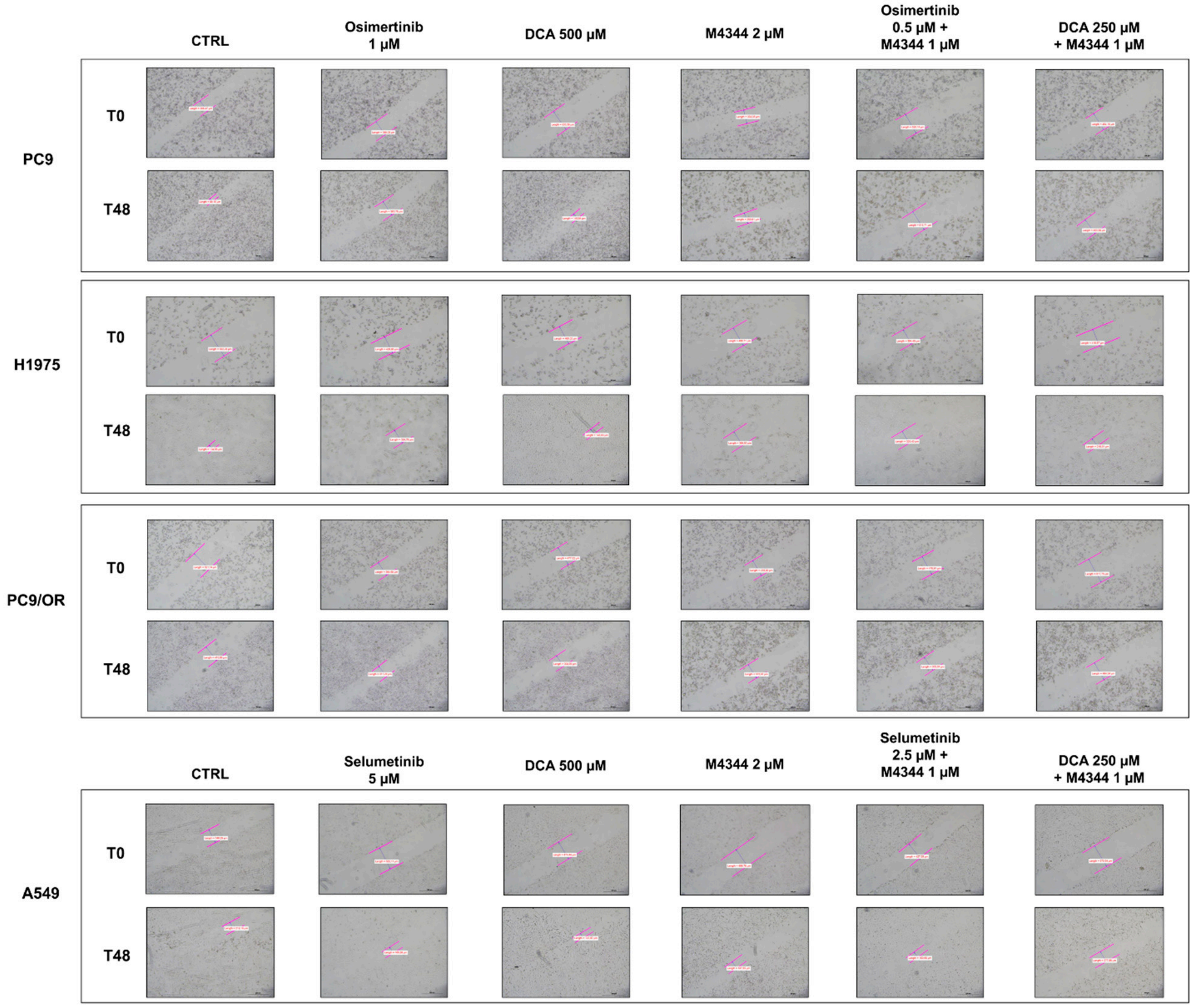

Figure S3. Representative live images of PC9, H1975, PC9/OR and A549 cells after scratch and treatments (osimertinib 1 µM, selumetinib 5 µM, DCA 500 µM or M4344 2 µM alone or in combination at half the dose) with a high-resolution microscope. Pictures (4X magnification) are captured fixing XY coordinates in order to measure the same gap point over time (T0-T48).

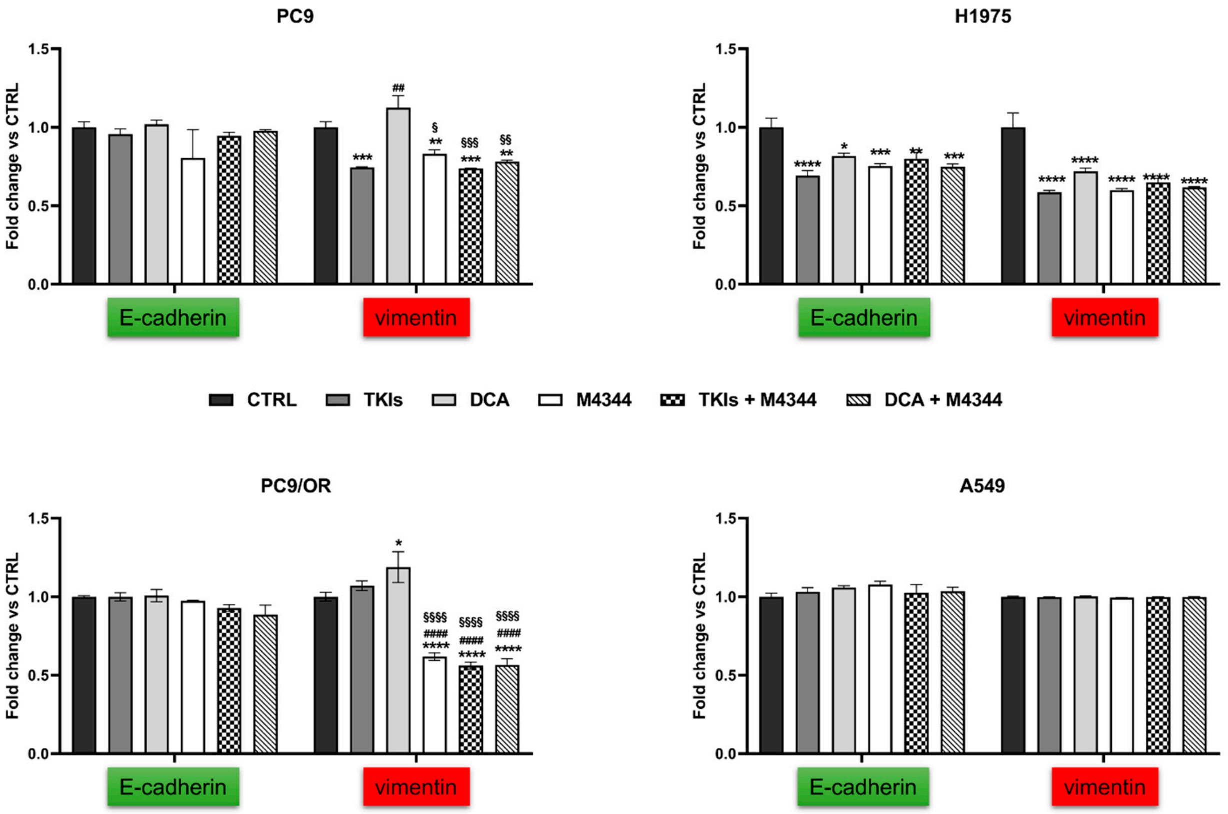

Figure S4. Quantitative IF analysis of e-cadherin and vimentin in PC9, H1975, PC9/OR and A549 cells after 48 h treatments (osimertinib 1 µM, selumetinib 5 µM, DCA 500 µM or M4344 2 µM alone or in combination at half the dose). Quantitative analysis of fluorescent intensity was expressed as fold change versus CTRL cells and expressed as mean ± SE. Statistical significance *$p < 0.05$, **$p < 0.01$, ***$p < 0.001$ and ****$p < 0.0001$ versus CTRL; $^{\#\#}p < 0.01$, $^{\#\#\#\#}p < 0.0001$ versus TKIs, $^{\S}p < 0.05$, $^{\S\S}p < 0.01$, $^{\S\S\S}p < 0.001$, $^{\S\S\S\S}p < 0.0001$ versus DCA. At least three independent experiments were performed.

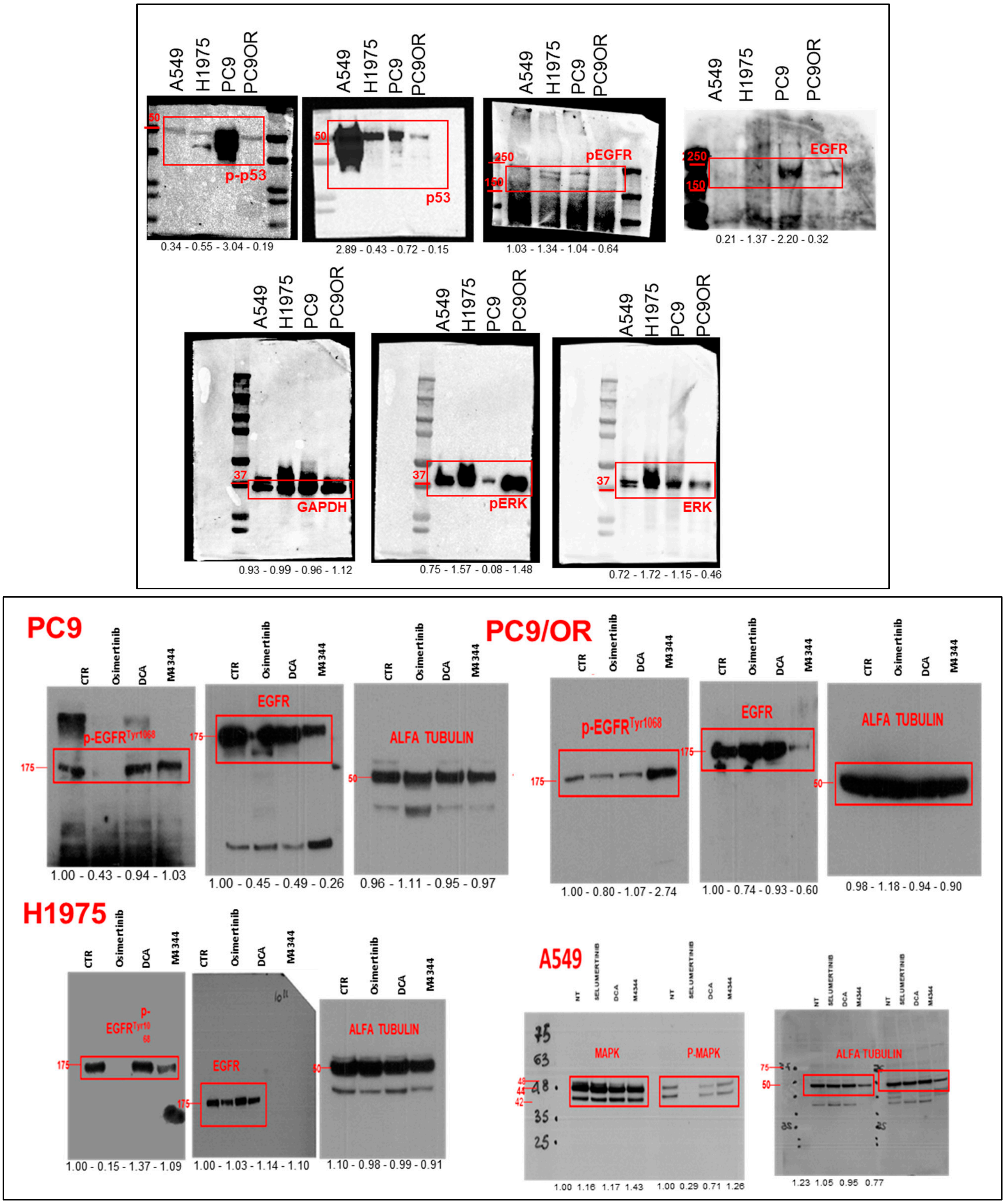

Figure S5. Original uncropped western blot images included in Figure 1A and 1B.

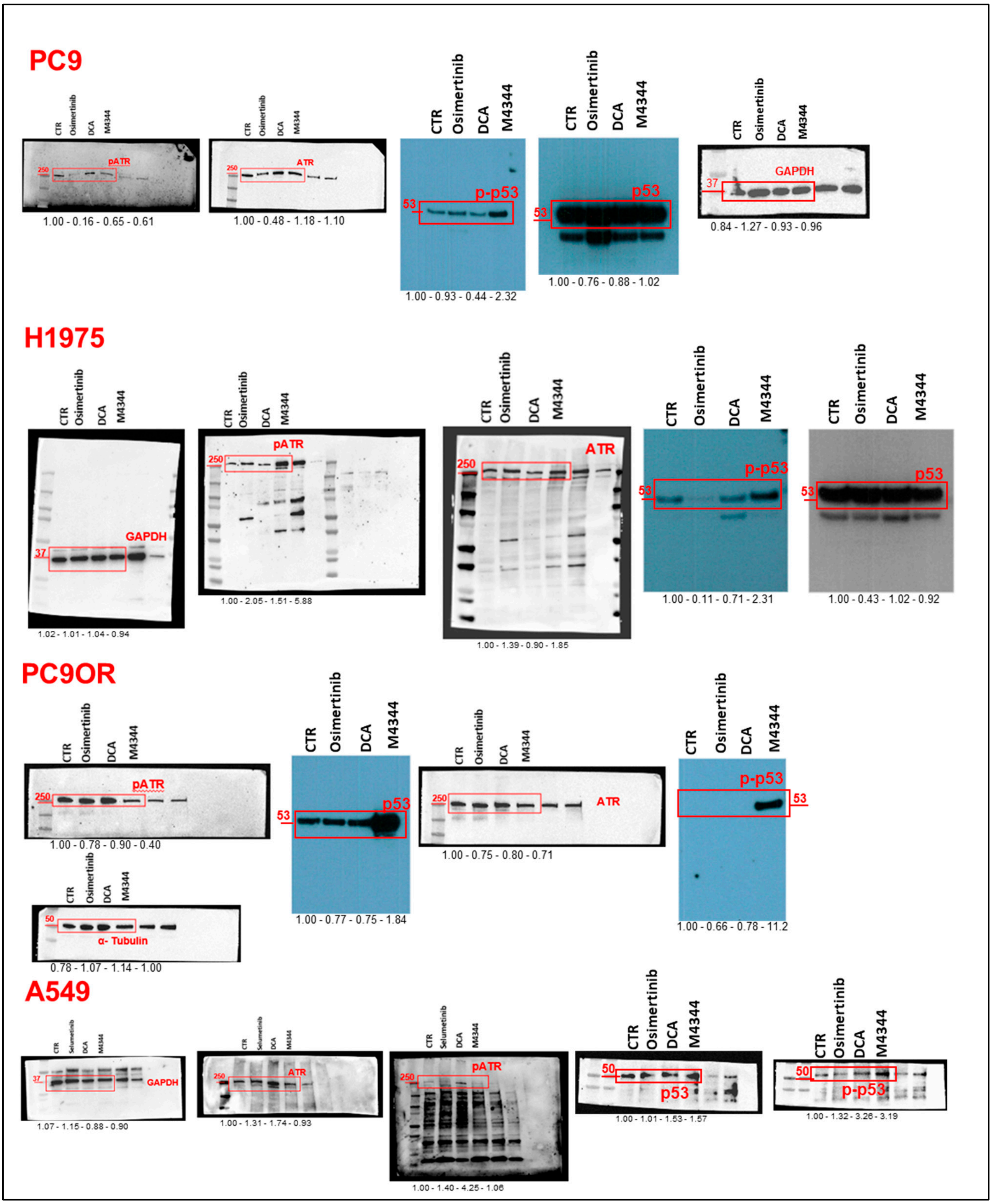

Figure S6. Original uncropped western blot images included in Figure 2A.

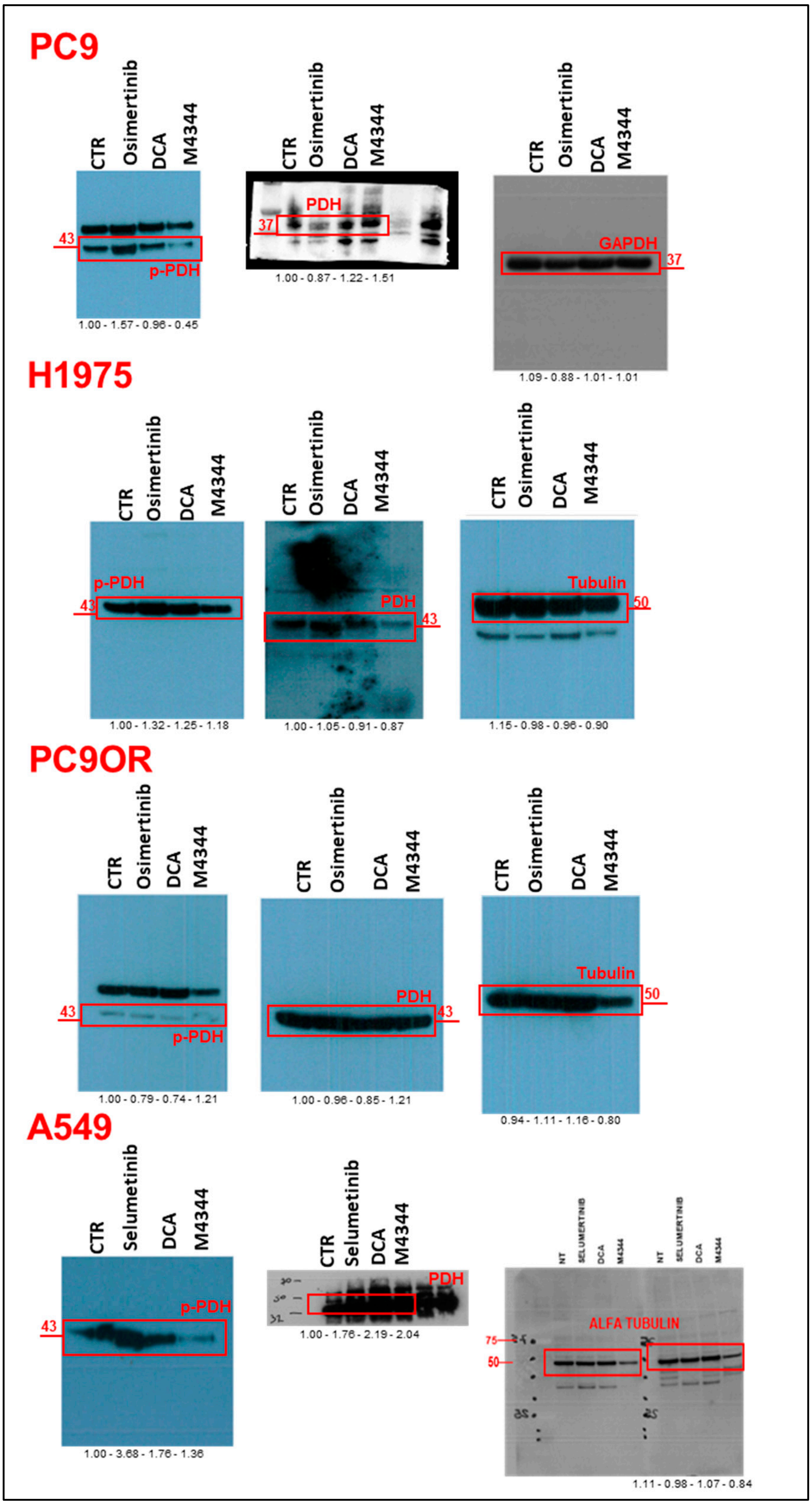

Figure S7. Original uncropped western blot images included in Figure 2B.

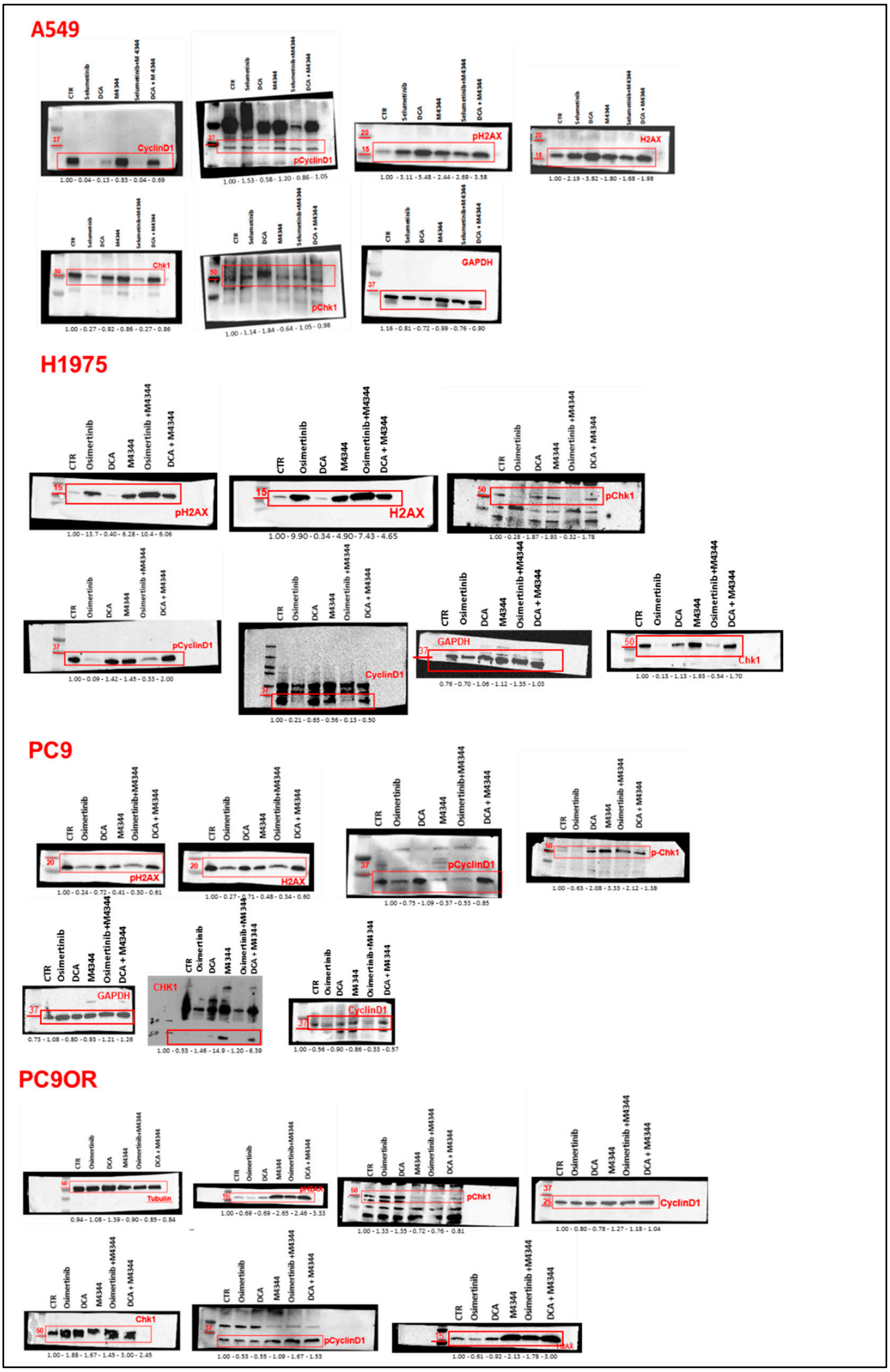

Figure S8. Original uncropped western blot images included in Figure 3B.

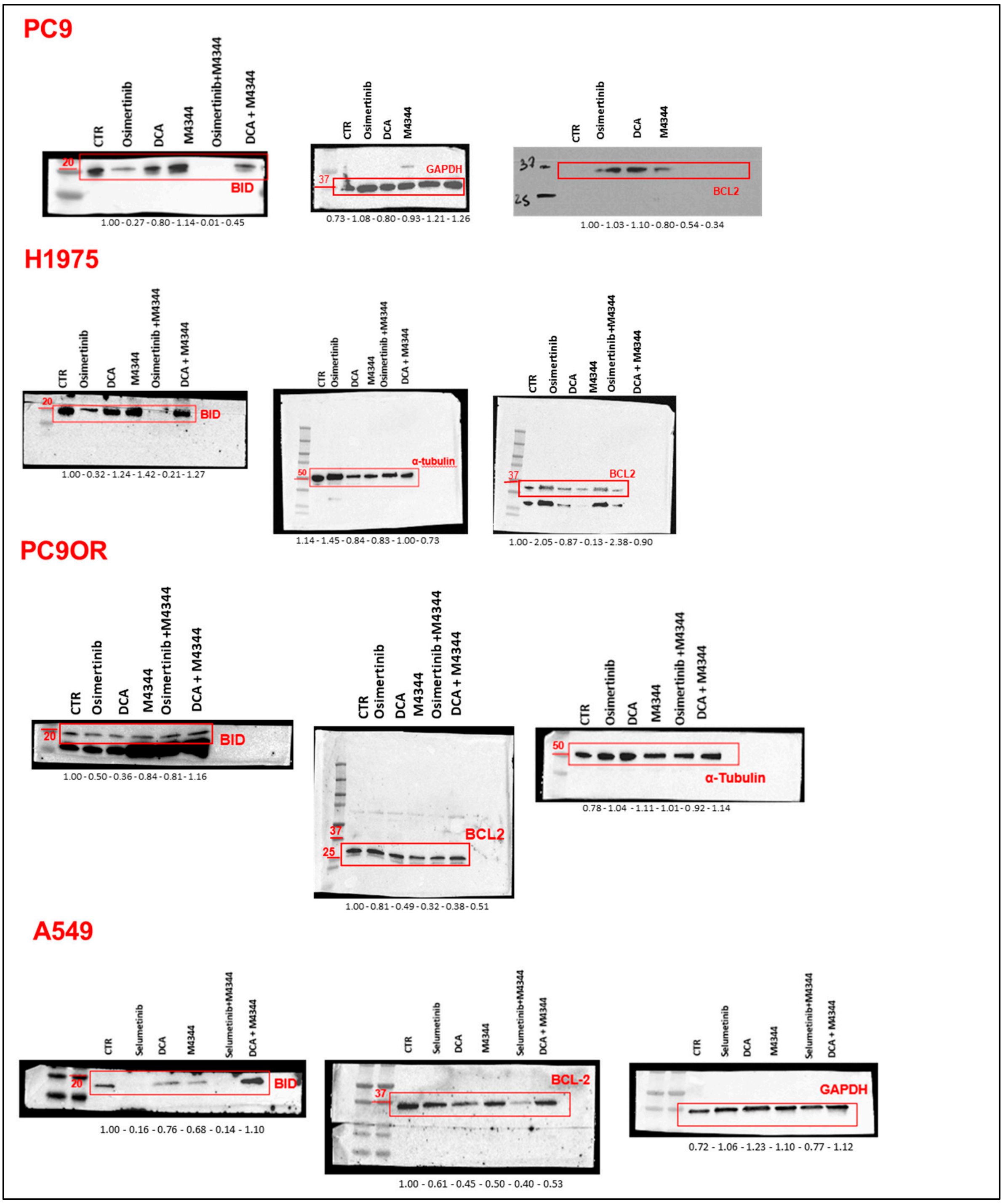

Figure S9. Original uncropped western blot images included in Figure 5B.
